# Initial results for patient setup verification using transperineal ultrasound and cone beam CT in external beam radiation therapy of prostate cancer

**DOI:** 10.1186/s13014-016-0722-7

**Published:** 2016-11-08

**Authors:** Anne Richter, Bülent Polat, Ingulf Lawrenz, Stefan Weick, Otto Sauer, Michael Flentje, Frederick Mantel

**Affiliations:** Department of Radiation Oncology, University of Wuerzburg, Josef-Schneider-Str. 11, 97080 Wuerzburg, Germany

**Keywords:** Prostate cancer, Transperineal ultrasound, IGRT, Setup verification, Cone beam CT

## Abstract

**Background:**

Evaluation of set up error detection by a transperineal ultrasound in comparison with a cone beam CT (CBCT) based system in external beam radiation therapy (EBRT) of prostate cancer.

**Methods:**

Setup verification was performed with transperineal ultrasound (TPUS) and CBCT for 10 patients treated with EBRT for prostate cancer. In total, 150 ultrasound and CBCT scans were acquired in rapid succession and analyzed for setup errors. The deviation between setup errors of the two modalities was evaluated separately for each dimension.

**Results:**

A moderate correlation in lateral, vertical and longitudinal direction was observed comparing the setup errors. Mean differences between TPUS and CBCT were (−2.7 ± 2.3) mm, (3.0 ± 2.4) mm and (3.2 ± 2.7) mm in lateral, vertical and longitudinal direction, respectively. The mean Euclidean difference between TPUS and CBCT was (6.0 ± 3.1) mm. Differences up to 19.2 mm were observed between the two imaging modalities. Discrepancies between TPUS and CBCT of at least 5 mm occurred in 58 % of monitored treatment sessions.

**Conclusion:**

Setup differences between TPUS and CBCT are 6 mm on average. Although the correlation of the setup errors determined by the two different image modalities is rather week, the combination of setup verification by CBCT and intrafraction motion monitoring by TPUS imaging can use the benefits of both imaging modalities.

## Background

During external beam radiation therapy (EBRT) of prostate cancer both interfractional and intrafractional movements of the prostate commonly occur. Changes in filling levels of bladder and rectum cause internal organ movements particularly in the anterior-posterior direction [1–3]. Image-guided radiotherapy (IGRT) provides the position of anatomical structures prior to treatment application. The most common technique of IGRT uses cone beam computed tomography (CBCT) based systems. CBCT based systems enable a reliable detection of interfractional target organ movements, however with the disadvantage of additional radiation exposure to the patient. Non-radiation based systems use ultrasound [4], optical surface detection [5], electromagnetic tracking [6] or MRI [7, 8]. Optical systems are suitable in situations where an external surface may act as a reliable surrogate for internal position or motion. This is not the case for localization of the prostate. Electromagnetic tracking makes use of transponders embedded in the tumor requiring a minimal invasive procedure for implantation. MR guided systems can visualize the soft tissue with an accuracy of 1–2 mm [9]. Limitation of MR based systems exist for patients with metallic implants and pacemakers. Ultrasound (US) imaging provides an alternative solution for treatment setup verification without an invasive procedure or additional imaging radiation dose. The technique of transabdominal US was evaluated by several groups [10–13] while the present study is focused on the transperineal setup of the US device. To date, no reports on transperineal US scanning for prostate localization are available. However, there is an ongoing controversial discussion on the precision of existing US systems [4].

The aim of this work was to validate detected set up errors using the transperineal ultrasound probe with Autoscan capability. The Clarity® system was evaluated in terms of accuracy in patient set up control. The setup error values detected by the CBCT system served as reference values.

## Methods

**Fig. 1:**
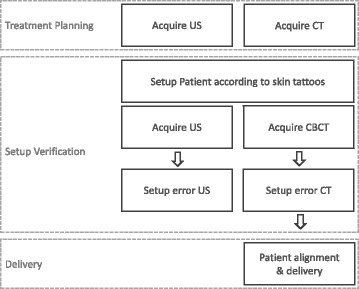
Schematic illustration of clinical workflow with setup verification using Conebeam CT and transperineal US

Setup verification was evaluated for ten patients who underwent EBRT for localized prostate cancer. The study was approved by the Ethics Committee of the University of Wuerzburg and all patients gave written informed consent. The patients were asked to reproduce their organ filling with an empty rectum and a filled bladder. The patients were advised to follow a drinking protocol and nutritional consultancy. The workflow of imaging for treatment planning and setup verification is illustrated in Fig. 1.

### Imaging and treatment planning

**Fig. 2:**
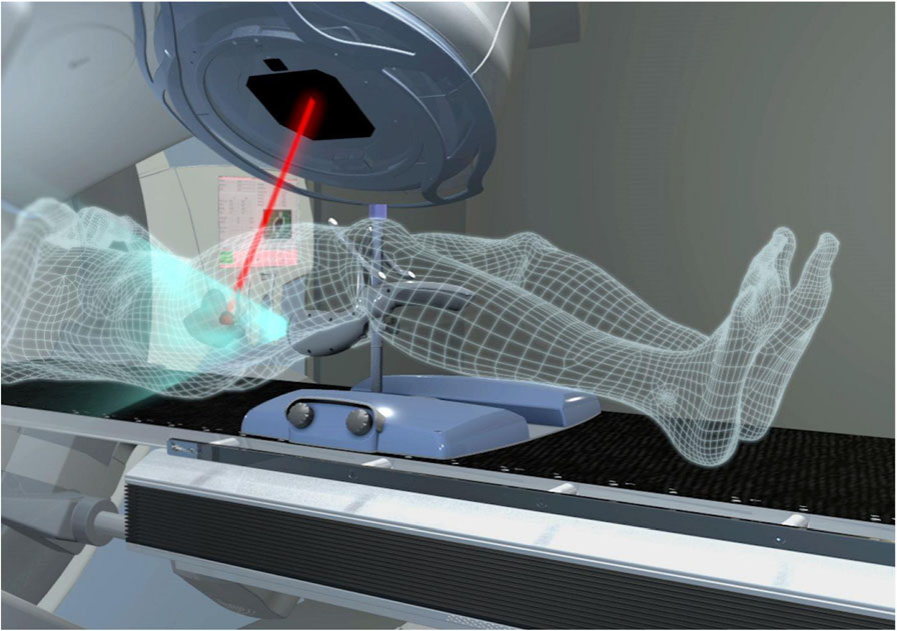
Schematic patient setup for transperineal ultrasound

For treatment planning, two image sets were acquired: a computed tomography (CT) scan (SOMATOM Sensation Open, Syngo CT, Siemens, Germany) and a 3D US reference scan (Clarity®, Elekta, Stockholm, Sweden). Both scans were performed in rapid succession in the identical supine patient position, stabilized with kneefix, foot support and the transperineal ultrasound (TPUS) probe in place. Figure 2 shows the schematic patient setup with the TPUS. For target volume and organ at risk (OAR) delineation patients also had an MRI scan with a dummy US probe. The Clarity Autoscan system has integrated a mechanically-scanned probe as described by Lachaine et al. [14].

Treatment was planned for an Elekta Synergy Platform® equipped with Agility Head and XVI Conebeam system (Elekta Oncology Systems, Crawley, UK). Pinnacle (Philips Radiation Oncology Systems, Milpitas, CA, USA) was used for treatment planning of a volumetric modulated arc technique with a simultaneous integrated boost (SIB). Total doses were D95 of 60Gy to the PTV and mean dose of 76.2Gy to the boost volume given in 33 fractions. The protocol of target volume definition and treatment planning was described in detail before [15, 16].

CT images, contours and treatment plan, were transferred to the Clarity planning workstation and registered to the US reference scan. The prostate was delineated based on TPUS images and defined as the Positioning Reference Volume (PRV). The PRV served as reference structure for setup verification of the patient position via TPUS in the treatment room. Both, scan acquisition and PRV delineation were performed by trained general users.

### Imaging and setup verification

Before treatment, the patient positioning was verified using the TPUS imaging directly followed by CBCT imaging. The setup error was recorded for both imaging systems for retrospective analysis. Compensation of the setup error and verification of treatment position was based on CBCT imaging. The planning CT and CBCT were registered automatically based on grey values followed by visual validation by the user. No rotational setup errors were determined, only translations were considered. CBCT imaging was performed daily for the first 7 fractions. Afterwards CBCT was acquired every third fraction. TPUS imaging was done for each fraction. The PRV was placed manually by the trained general user in the US image set. For each patient, setup verification with both image modalities was available in 15 fractions.

### Comparison of prostate localization

First, setup errors were analyzed separately for each dimension to find out if there is any predominant direction with a large deviation. Evaluation in lateral, vertical and longitudinal direction corresponded to left-right, anterior-posterior and superior-inferior direction, respectively. The 3D vectors (Euclidean distance) of the setup errors were then compared. The overall mean was calculated from the absolute differences between CBCT and TPUS. The difference between CBCT and TPUS prostate localization was analyzed in a correlation and Bland-Altman plot [17]. The limits of agreement (Mean ± 2*SD) indicate if the two methods are interchangeable and if the difference between the upper and lower limit is within a clinically acceptable range. Significance of the differences among TPUS imaging and CBCT was tested by the paired two-sided Student’s *t*-test.

## Results

In total 150 TPUS- and CBCT scans were acquired sequentially and analyzed to verify the treatment position. The obtained setup errors from the TPUS and CBCT imaging were compared. Results are listed in Table 1.

**Table 1:** Summary of setup errors: mean, standard deviation, median and range

Dimension	Mean ± STD	Median	Range
Lateral			
US imaging	−0.4 ± 3.8	−0.3	−12.6 → 10.3
CBCT imaging	1.1 ± 3.6	1.4	−7.3 → 12.1
Absolute difference of US and CBCT	2.7 ± 2.3	2.5	0 → 10.3
Vertical			
US imaging	0.8 ± 4.1	1.0	−12.6 → 11.0
CBCT imaging	0.3 ± 3.3	1.2	−12.0 → 7.7
Absolute difference of US and CBCT	3.0 ± 2.4	2.5	0 → 11.8
Longitudinal			
US imaging	−0.5 ± 4.4	−0.8	−11.9 → 11.3
CBCT imaging	0.9 ± 3.2	0.6	−10.3 → 9.7
Absolute difference of US and CBCT	3.2 ± 2.7	2.6	0 → 16.5
3D			
US imaging	6.0 ± 2.9	6.3	0.8 → 16.9
CBCT imaging	5.4 ± 2.7	4.9	0.6 → 13.3
Absolute difference of US and CBCT	6.0 ± 3.1	5.6	1.1 → 19.2

In units of mm

### Setup error distribution of TPUS and CBCT imaging

For CBCT imaging, setup errors ranged from −7.3 to 12.1 mm, from −12.0 to 7.7 mm and from −10.3 to 9.7 mm in lateral, vertical and longitudinal direction, respectively. The absolute mean setup error for CBCT imaging was (1.1 ± 3.6) mm, (0.3 ± 3.3) mm and (0.9 ± 3.2) mm in lateral, vertical and longitudinal direction, respectively.

For US imaging, a larger setup error range was measured with ranges of −12.6 to 10.3 mm, −12.6 to 11.0 mm and −11.9 to 11.3 mm in lateral, vertical and longitudinal direction, respectively. The absolute mean setup errors for US imaging were (−0.4 ± 3.8) mm, (0.8 ± 4.1) mm and (−0.5 ± 4.4) mm in lateral, vertical and longitudinal direction, respectively. In 70 % and 49 % of all TPUS and CBCT sessions the 3D setup error of 5 mm was exceeded.

### Comparison of TPUS and CBCT imaging

**Fig. 3:**
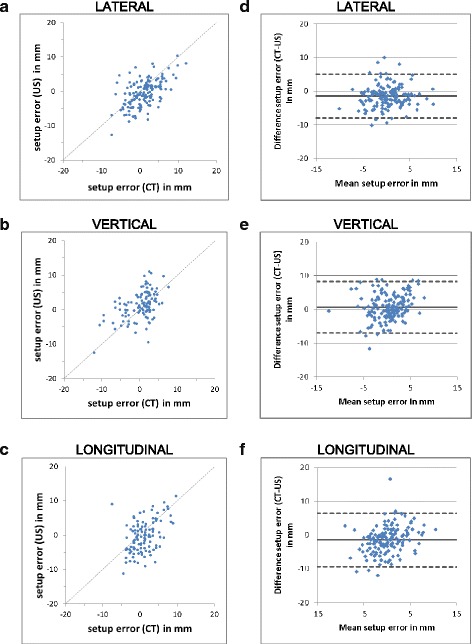
Setup errors measured with US and CBCT imaging shown in correlation plots with line of equality (**a**, **b**, **c**) and Bland-Altmann plots (**d**, **e**, **f**)

The setup errors of TPUS imaging as a function of CBCT imaging are plotted in Fig. 3a–c for each dimension. There is only poor agreement of the data points with the line of equality. Pearson correlation coefficients of −0.57, 0.49 and 0.49 were calculated in lateral, vertical and longitudinal direction, respectively. The strength of linear correlation was moderate in all direction. The correlation between CBCT and TPUS setup errors was significant in all direction (*p* < 0.01) only. By the two-sided paired Student’s *t*-test, there was no significant difference between TPUS and CBCT in vertical direction (*p* = 0.07) whereas there was significant difference in lateral and longitudinal direction (*p* < 0.001).

The difference between setup error measurements as a function of the mean of both values is shown in a Bland-Altmann analysis in Fig. 3. The upper and lower limits of agreement were 5 mm / −8 mm, 8.2 mm / −7.1 mm and 6.5 mm / −9.4 mm in lateral, vertical and longitudinal direction, respectively.

**Fig. 4:**
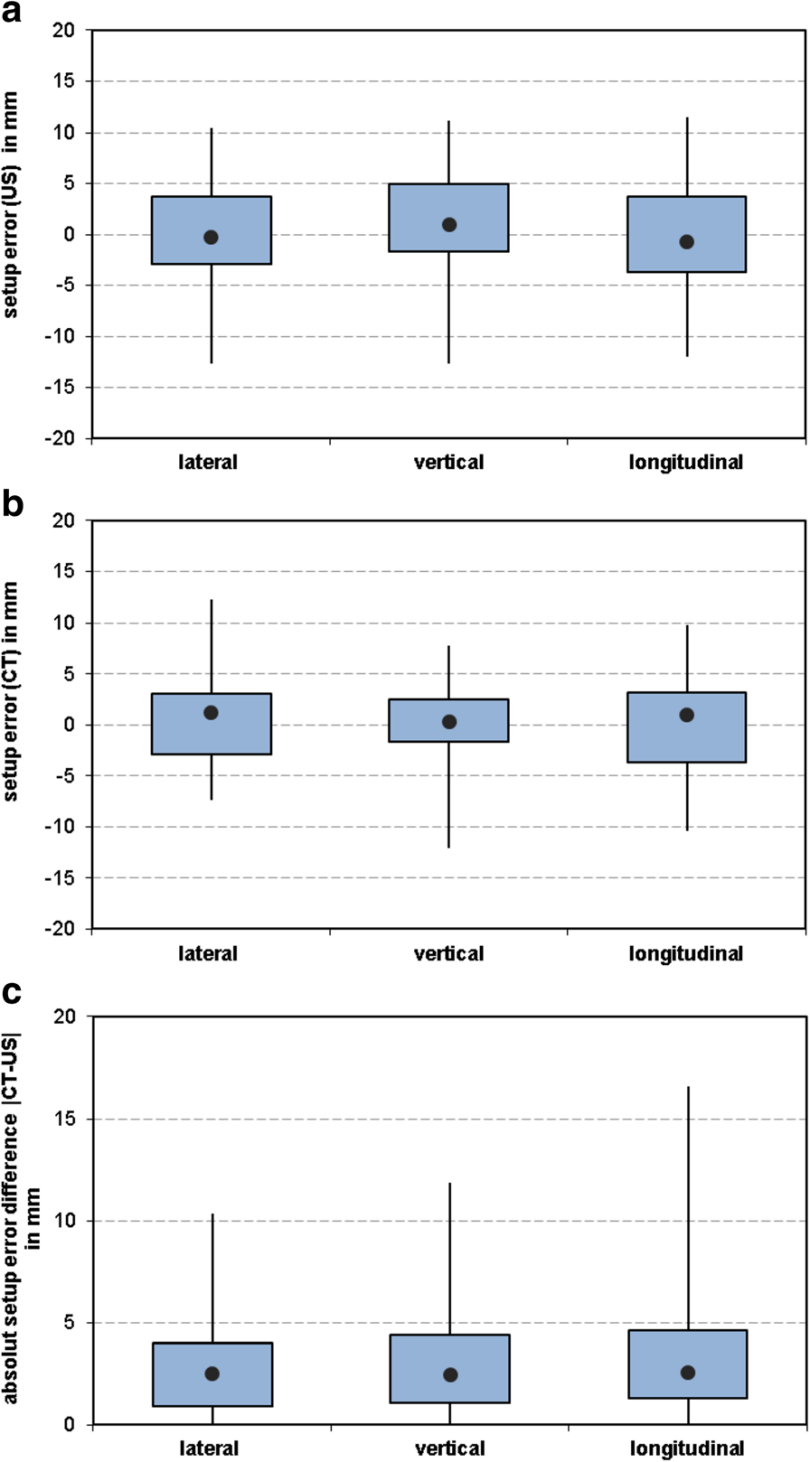
Boxplots of setup errors for (**a**) US, (**b**) CBCT imaging and (**c**) CT-US in lateral, vertical and longitudinal dimension

The distribution of setup errors is summarized in boxplots in Fig. 4 for each dimension and imaging modality. The average distance between CBCT and TPUS was (2.7 ± 2.3) mm, (3.0 ± 2.4) mm and (3.2 ± 2.7) mm in lateral, vertical and longitudinal direction, respectively. The boxplot in Fig. 4c shows the distribution of absolute distance between CBCT and TPUS in lateral, vertical and longitudinal direction. Similar values were calculated for the median deviation with 2.5 mm, 2.5 mm and 2.6 mm in lateral, vertical and longitudinal direction, respectively. As shown in Fig. 4c, the largest discrepancies between TPUS and CBCT were observed in longitudinal dimension with a median deviation of 2.5 mm and a maximum deviation of 16.5 mm. In lateral dimension, the lowest deviation between both imaging systems was observed with a median difference of 2.5 mm and a maximum deviation of 10.3 mm. The minimum observed discrepancy between CT and TPUS setup errors in 3D was 1.1 mm, while the maximum was 19.2 mm. The resulting mean deviation in 3D was (6.0 ± 3.1) mm with a median deviation of 5.6 mm. The difference between TPUS and CBCT was larger than 5 mm in 58 % of all monitored treatment sessions. Differences larger than 10 mm occurred in 11 % of the monitored treatment sessions.

## Discussion

### Comparison of IGRT techniques

Mean differences between TPUS and CBCT determined shifts of 2 to 3 mm for each dimension were observed. The resulting Euclidean distance was 6 mm on average. However, for individual setups differences up to 20 mm occurred. The difference between TPUS and CBCT was larger than 5 mm and 10 mm in 58 % and 11 % of all monitored treatment sessions.

Robinson et al. evaluated the differences between CBCT and transabdominal US imaging. They reported a mean discrepancy of 10 mm while our evaluation resulted in an average distance of 6 mm [12]. Other studies compared the accuracy of setup verification for implanted fiducial markers and US in each cartesian direction [11, 13]. The mean differences in setup errors were up to 2–3 mm which is comparable to our results with a median deviation of 2.5–2.6 mm deviation.

In our study, larger differences were observed in longitudinal direction (3.2 ± 2.7) mm while smaller deviations were seen in lateral direction (2.7 ± 2.3) mm. This is in agreement to the results published by other groups [11, 13]. The Bland-Altmann analysis resulted in limits of agreement of 8 to 10 mm covering 95 % of the measurements. The largest limit of agreement was calculated in longitudinal direction (6.5 mm/−9.4 mm). This is in agreement to the results published by van der Meer et al. who obtained a limit of agreement of 10 mm in longitudinal direction [13]. In contrast, Robinson et al. observed the greatest disparity between CT and transabdominal US in the vertical direction [12].

Discrepancies between TPUS and CBCT of 5 mm and more occurred in 58 % of our monitored treatment sessions. Discrepancies of 10 mm were exceeded in 11 %. Again, Robinson et al. observed larger deviations: 5 mm were exceeded in 89 % and 10 mm were exceeded in 42 % of the measurements [12]. Van der Meer et al. showed that in 56 % of all patients the difference in at least one direction was larger than 5 mm (>10 mm for 11 %) [13]. In the present study, the distribution of setup errors showed a larger range for TPUS (0.8 - 16.9 mm) than for CBCT (0.6 - 13.3 mm). The mean and standard deviation were slightly smaller for CBCT imaging (TPUS: 6.0 ± 2.9 mm and CBCT: 5.4 ± 2.7 mm). The absolute difference in 3D between both imaging modalities was (6.0 ± 3.1) mm with a median deviation of 5.6 mm. Subsequently, setup differences between TPUS and CBCT are 6 mm on average. These preliminary results will be examined in a larger patient population in our further research. Some groups report that the accuracy of US is comparable to fiducial marker based localization [11, 13] while another group concluded that the US fails to deliver an acceptable level of geometric accuracy with regard to prostate localization [12].

In comparison to US, external localization systems like MRI-guided IGRT and electromagnetic tracking systems achieve a geometric accuracy of 1–2 mm [9, 18]. Mayyas et al. compared the interfraction setup errors between different localization techniques (kV, CBCT and US imaging and electromagnet transponders) [19]. They found that that the kV and CBCT shifts are comparable to that of US.

Image acquisition for CBCT is a user independent technique, which is widely used for setup verification during radiation therapy for prostate cancer [20]. However, CBCT leads to additional patient dose. That is admittedly low in comparison to the treatment related dose, but is not fully negligible in the patient cohort of prostate EBRT with a relatively favorable prognosis. The CBCT is reasonably not used for setup verification in every treatment session. Whereas, a daily use of the US imaging is very well possible and can potentially detect a disadvantageous bladder and rectum filling earlier. This information can be incorporated in the treatment workflow by asking the patient to adapt bladder or rectum filling.

Besides setup verification, the transperineal setup of the US device additionally offers the possibility of intrafraction target monitoring and possible motion management in the future. A first proof of principle experiment of US based motion tracking was performed in a phantom study by Schwaab et al. with promising results but the need of further research [21]. Colvill et al. studied the dosimetric impact of tracking and gating to account for intrafraction motion during prostate radiotherapy [22]. O’Shea et al. reviewed the applicability of intrafraction monitoring with US for prostate and other treatment sites [23]. Until further results on the practicability and accuracy of TPUS are available, certainly the combination of setup verification by CBCT and intrafraction target organ and OAR motion monitoring by TPUS imaging can use the benefits of both imaging modalities.

### Limitations

One limitation is that no rotational errors were determined for prostate positioning. The US imaging and registration offers translational errors only. For setup verification of patients with prostate cancer, we correct for translational errors according to our protocol due to practicability and patient stability. Effects of rotations and translations of the prostate should be taken into account during target delineation and margin definition. The correction of rotational errors becomes more important for the hypofractionated radiotherapy treatment of prostate cancer and treatment of large, complex target volumes or simultaneous, single isocenter irradiation of multiple targets [24]. The correction of rotational errors can induce substantial secondary patient displacements if the patient is not immobilized sufficiently [25].

Another limitation of our study is the small number of patients. The results will be examined in a larger patient population in further research.

The applicability of US for prostate localization is discussed controversially in the literature due to its uncertainties like user variability (scanning variability and registration variability). Different probe pressures during image acquisition can lead to unreproducible target organ movements [4, 11–13]. The influence of the probe pressure on the reproducibility is still an issue of concern [4, 10, 13, 16]. The advantage of the Clarity system is the Autoscan probe decreasing the scanning variability. As we found out in a recently published study, the transperineal probe setup shifted the penile bulb in cranial direction and thereby increased the dose to the penile bulb [16]. However, in that study, no relevant changes in the prostate or PTV volumes were observed and organ motion of the prostate was only seen to a minor extent mainly in the superior direction. [26]. This seems to substantiate the assumption that probe pressure induced organ motion is reduced to a minimum by the transperineal scanning approach compared to transabdominal US. The variability of probe pressure is further reduced within the TPUS Clarity® system by probe fixation and a height scale as well as the display of the current and planned probe position in the software. Nonetheless, as low pressure of the TPUS probe as feasible to still achieve appropriate image quality is recommended for limitation of structure shifts and further reduction of placement variability.

User variability is influenced not only by scanning, but also by image registration and the manual placement of the Reference Positioning Volume. For the transabdominal US approach, van der Meer et al. reported a user variability of 2–3 mm – including scanning and matching variability [13]. Van der Meer determined the intra- and interoperator variability for transabdominal US. In their study, the intraoperator match variability was in the range of 1 mm and the interoperator variability was in the range of 1.3 - 1.8 mm. Because the accuracy of US is highly influenced by the user more effort is required to standardize US imaging compared to CBCT or portal imaging, as van der Meer already stated [13]. This certainly includes a comprehensive employee training but also improvements from the technical side are warranted like automated image registration and segmentation.

## Conclusion

The evaluation of setup verification with TPUS and CBCT showed setup differences of 6 mm on average. Due to its large user dependency more effort is required to standardize US imaging compared to CBCT or portal imaging. At the moment, the combination of setup verification by CBCT and intrafraction motion monitoring by TPUS imaging can use the benefits of both imaging modalities.
